# PDE2, a component of the NO/cGMP signalling in the hippocampus

**DOI:** 10.1186/1471-2210-11-S1-P63

**Published:** 2011-08-01

**Authors:** Isabel Schönle, Angela Neitz, Thomas Mittmann, Doris Koesling, Evanthia Mergia

**Affiliations:** 1 Institute of Pharmacology, Medical School, Ruhr-University Bochum, Bochum, Germany; 2 Institute of Physiol. & Pathophysiol., Univ. Med.-Center of the Joh.-Gutenberg-Univ. Mainz, Germany

## Background

NO/cGMP-mediated signal transduction is involved in synaptic plasticity in various brain regions. NO effects are transduced by the NO receptor guanylyl cyclase (NO-GC) that exists in two isoforms, NO-GC1 and NO-GC2, with indistinguishable regulatory properties. Mice deficient in either NO-GC1 or NO-GC2 revealed that both NO-GC isoforms are required for LTP indicating the existence of two separated NO/cGMP pathways. Recently, we demonstrated a presynaptic role of NO/cGMP in facilitation of glutamate release and indentified eNOS and NO-GC1 as the participating enzymes. Yet, the involved cGMP-hydrolysing phosphodiesterases (PDE) remained unknown.

## Results

Here we demonstrate that PDE2 accounts for 50% of cGMP-hydrolysing activity in hippocampal homogenates. In hippocampal slices of WT, NO-GC1 and NO-GC2 KO mice, PDE2 inhibition increased NMDA-induced cGMP levels.

## Conclusion

This suggests PDE2 as a component of both NO-GC1- and NO-GC2-mediated signalling pathways. Moreover we analyzed the physiological role of the PDE2 on glutamatergic transmission in the hippocampal CA1 region by single-cell recordings in acute slices.

